# A Law of Heridity, &c.

**Published:** 1889-03

**Authors:** 


### A LAW OF HERIDITY, OR POSSIBLY, MATERNAL IMPRESSIONS.

Read before the Dallas County Medical Society and by a resolution, voted to be published.

From the earliest ages the surroundings of the ancient woman have been attributed with influencing the development of the fœtus. It antedates the successful experiment in holy writ, where the God of Israel instigated Jacob to cheat his father-in-law, Laban, out of all the desirable increase of his flocks and herds. The Greeks anticipated Darwin in recognizing the propriety of the law of the survival of the fittest, and enacted a decree to destroy all deformed and sickly children at birth, to ensure a physically perfect race. They surrounded prospective mothers with paintings, statuary and other adjuvants to influence mental development in the fœtus, and who can say how much they are indebted to such practices for the place they hold in the world of art? However, the spirit of modern research is no longer shackled by the theories and opinions of the past. Outside of theological circles the very fact that ideas are ancient, renders them liable for arraignment at the bar of investigation; for how could men in the dawn of creation perceive truth clearer than their decendants, who can focus the light of ages to aid them in the search? Tho' science has never immolated an investigator at the stake, for his opinions, it has had its full share of superstitions, and there has been no department of research more prolific than the question of fœtal development. Its laws are obscure, partly from the fact that for data science largely depends upon that class in society who, on account of their circumscribed lives, longest cling to tradition and superstition. That there are laws that govern such things, is but a carollary to the fact that all creation is governed by law. Medical men have been so annoyed by the superstitions clinging about the subject of maternal impressions and heredity, they have refused to investigate while more practical qestions were clamoring for solution. They have followed the Ariadne thread of science thro' the labyrinth of human suffering solely to give battle to the Microbe in the citadel.

Unlike the philosophers of old who despised practical application of scientific truth, modern research has had for its object the improvement of the present existence. Plato, the philosopher of ecclesiasti cism formulated theories, and if the facts did not sustain the theorem so much the worse for the facts. Upon Aristotle's method of reasoning is based every modern science. He collected facts, and from them deducted general laws. His conclusions were sometimes erroneous from insufficient data, but the process tho' slow, is our only means of arriving at truth. The observations of a single individual must necessarily furnish meagre material upon which to base a law on so obscure a question, but never having seen any literature upon the subject, that must be my only resource.

In a large majority of cases the first child born shortly after marriage, resembles the father. The second is more likely to be a repro-production of the mother. After this, the parental type is mingled and irregular. Should the birth of the first child be delayed more than a year after marriage, the chances are against the impress of the father and in favor of the type of the mother. There is a popular belief that children take their physical type from one parent and their mental attributes from the other; but such is not the case. Parental traits may be mingled in complex forms, but a distinctly physical type is generally found in connection with the mental characteristics which distinguish that side of the family. This fact accords perfectly with the position science takes in regard to the close relation between the mental and physical. In society organized like our own, largely upon individual freedom, parties assume the marriage relation more or less of their own free wiil. It is not a matter of judgement, but of feeling, and the impress of the father is greater when his ascendency is at the maximum. When the glamour of the new relation has been dispelled by familiarity, and life is reduced to prosaic realities, the mother's individuality asserts itself, and the second child is an accentuation of her type. After this she adjusts herself to her surroundings, looks at life more philosophically, and the third child may return to the type of the father. Sometimes the father is strongly marked in the first child and the likeness gradually disappears, in subsequent additions to the family. The reverse of this has never been observed by the writer. An illustration of the part played by the mother's feelings in reproduction came under observation, where a reversal of conditions went to prove the rule. A young, pretty, thoroughly selfish woman married a homely, elderly man, from mercenary considerations. The first child was an exact reproduction of the mother. The mother was one of those quiet individuals that improve upon acquaintance, and it was not long before she was as thoroughly impressed as her selfish nature was capable of being, and the likeness of the second to the father was as marked as that of the first to the mother. Succeeding increase in the family circle naturally reverted to the type of the mother. Sex seems to play no part in deciding the resemblance to parents.

The facts here given are correct, tho' the theory of their cause may be wide of the truth. Could statistics be collected from those countries where feeling plays no part in the marriage contract, and the facts found to be the same, the theory would be exploded. If the hypothesis is correct and the mother's feelings for the father leave their impress on the progeny, could not other causes exciting the mother's feelings reach the fœtus? If the normal course of nature is superceded, other laws assert themselves or variously modify the original tendency. Possibly other emotions besides those registered by the affections may reach the fœtus and leave their impress. Two interesting cases came under the writer's observation. A child was born the exact image of a gentleman who sat opposite the mother at mealtime, and was not her husband. There was no question of paternity, for the woman was as pure as Cæsar's wife should have been. The young man was readheaded and freckled face and there were no such characteristics on either side of the family to be accounted for by the law of atavism. Neither was there any reason to believe the woman harbored any feeling toward her red-headed vis-a-vis she would not have been perfectly willing to acknowledge to her husband. It seemed to be an illustration of Jacob's theory of ring streaked and striped poles for the cattle. The other case, a boy, had every characteristic of the negro race so far as form and feature were concerned. His hair was red, and curled on the flat in regular negro kinks. His complexion was of the peculiarly fair kind that generally accompanies a read head, indicating not the slightest taint of negro blood. The writer knows nothing of the facts connected with the gestation of this case.

These things can hardly be ascribed to accident, because the scientific world of to-day has banished providence and accident as factors in the law of cause and effect. The weight of scientific evidence is against the emotions of the mother affecting the embryo after conception has taken place, but the facts given seem to indicate that at some time in ante natal life the mother's emotional condition leaves its impress on the little life that is being created by laws as positive as those that evolve solar systems from their gaseous elements. These things are not understood and 'tis better to exercise toward them scientific tolerance than to permit the spirit of bigotry to close the door of investigation. He who is not willing to give up to day the position he defended yesterday, when he finds the weight of evidence against him, loves himself better than he loves truth.—*Texas Courier Record.*

